# Efficacy of different doses of dexmedetomidine as a rapid bolus for children: a double-blind, prospective, randomized study

**DOI:** 10.1186/s12871-018-0562-0

**Published:** 2018-08-07

**Authors:** Fang Chen, Chengyu Wang, Yi Lu, Mengmeng Huang, Zhijian Fu

**Affiliations:** 1 Department of Pain Management, Shandong Provincial Hospital affiliated to Shandong University, 324 Jingwu Road, Jinan, 250021 China; 2 Department of Anesthesiology, Critical Care and Pain Medicine, The 2nd Affiliated Hospital & Yuying Children’s Hospital of WenZhou Medical University, Wenzhou, China

**Keywords:** Dexmedetomidine, Children, Rapid injection, Emergence agitation

## Abstract

**Background:**

Dexmedetomidine (DEX), a highly sensitive α_2_-adrenoceptor agonist that possesses anxiolytic, sedative, and analgesic effects, has been documented as a preventative and treatment for emergence agitation (EA). The therapeutic should be given as a loading dose that is infused during a 10 min period, but if a rapid bolus injection is deemed to be hemodynamically appropriate, it would be a more opportune route of administration. So we studied the efficacy of different doses of DEX as a rapid bolus for children to prevent and treat EA.

**Methods:**

One hundred patients were enrolled and randomly divided into five groups: the control group (group D_1_), the 0.25 μg/kg DEX group (group D_2_), the 0.5 μg/kg DEX group (group D_3_), the 0.75 μg/kg DEX group (group D_4_), and the 1 μg/kg DEX group (group D_5_). Heart rate (HR), mean blood pressure (MBP) and blood oxygen saturation (SaO_2_) were recorded immediately before the study drug injection (baseline) and every minute for 5 min thereafter and at the time points of the skin cut and hernial sac pull. EA and pain were assessed in the post -anesthesia care unit, and any complementary medicine and adverse events were recorded too.

**Results:**

The incidence of EA was significantly decreased in group D_4_ and group D_5_ compared with D_1_.All groups exhibited similar baseline HR and MBP. After administered, HR and MBP were significantly decreased in all DEX group compared with group D_1._In groups D_3_, D_4_ and D_5_, the minimal HR was decreased significantly compared with the groups D_1_ and duration time of minimal HR significantly prolonged in group D_5_, but no patient needed treatment. As the dosage increased, the recovery time was significantly prolonged. There were no significant differences in occurrence time of minimal HR, the incidence of complementary medicine and adverse events among groups.

**Conclusion:**

Rapid intravenous injection (IV) bolus administration of 0.75 and 1.0 μg/kg of DEX could improve the recovery profile by reducing the incidence of EA in children. Although its use resulted in a transient decreases in HR and MBP, DEX was clinically well-tolerated in children.

**Trial registration:**

No. ChiCTR-IPR-17010658. Registered 17 February 2017.

## Background

Dexmedetomidine (DEX), a highly selective α_2_-adrenoreceptor agonist, offers anxiolytic, sedative, and analgesic effects with negligible respiratory depression [1–3].DEX has been approved for use in adults, but the US Food and Drug Administration has not approved the drug for children. However, DEX has been documented in pediatric patients as a premedication, a sedative in the pediatric intensive care unit, in conjunction with inhaled anesthetic agents, and as a therapeutic for the prevention and treatment of emergence agitation (EA) following general anesthesia [4–6]. EA, particularly in children, presents a great challenge to good patient care [7, 8]. While 2-, 5-, or 10-min DEX infusions lower the occurrence of EA in pediatric patients [9, 10], it is typically not convenient to administer a bolus infusion in the clinical area of an elevated-turnover work-intensive pediatric anesthesiology unit. A quickly administered bolus injection, if deemed to be hemodynamically appropriate, would be a more opportune route of administration to avert and treat EA. Jooste EH et al. [11] had observed that rapid IV bolus administration of DEX in 12 children who underwent heart transplants was clinically well-tolerated, although its use resulted in a transient but significant increase in systemic and pulmonary blood pressure and a decrease in heart rate (HR). In addition, Hauber JA et al. [12] documented that fast IV bolus administration of 0.5 μg/kg DEX improved pediatric patients’ recuperation profile by lowering the occurrence of EA. Although a statistically significant change in hemodynamics was observed, no patient required intervention for hemodynamic changes. However, the aforementioned studies used either a small sample study or only one dose. DEX is often used to avert and treat postoperative agitation in dosages of 0.25–1 μg/kg, and many clinical institutions now commonly administer DEX as a rapid (under 5 s) IV bolus. Therefore, our study examined the efficacy of different doses of DEX as a rapid bolus for prevention and treating EA in pediatric patients.

## Methods

### Design and setting

We conducted a double-blind randomized controlled trial in 100 patients in the 2nd Affiliated Hospital & Yuying Children’s Hospital of WenZhou Medical University.

### Patient selection

According to the preliminary experimental results, two groups with the smallest difference in the five groups were analyzed with a = 0.05, b = 0.2, and the difference between groups was 0.4.The calculated sample size was 16 cases per group. Considering the 20% sample exfoliate rate, we need 20 cases per group, a total of 100 cases sample size.

This clinical trial was registered at http://www.chictr.org.cn (No. ChiCTR-IPR-17010658), and conducted from April 2017 to October 2017. After obtaining informed written consent from parent of each child, 100 ASA I or II pediatric patients, aged 3–7 yr., were randomly (Random number method) enrolled into five groups: the control group (group D_1_), the 0.25 μg/kg DEX group (group D_2_), the 0.5 μg/kg DEX group (group D_3_), the 0.75 μg/kg DEX group (group D_4_), and the 1 μg/kg DEX group (group D_5_). Inclusion criteria were: normal intelligence and normal liver and kidney function, scheduled for elective inguinal hernia repair surgery, entering the operating room by themselves without parents, and no history of an allergy to anesthesia.

**Fig. 1:**
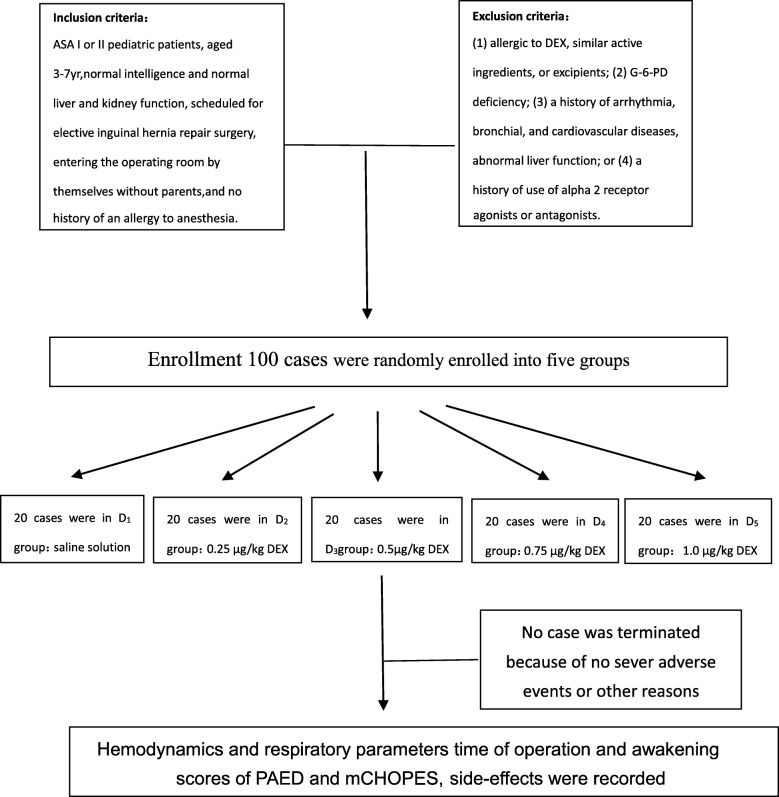
The flow chart of the study

Patients were excluded if they met any of the following criteria: (1) allergic to DEX, similar active ingredients, or excipients; (2) G-6-PD deficiency; (3) a history of arrhythmia, bronchial, and cardiovascular diseases, abnormal liver function; or (4) a history of use of alpha _2_ receptor agonists or antagonists (Fig. 1).

### Medication

No premedication was administered. A peripheral venous catheter was inserted approximately 2 h prior to surgery (while in the unit). EMLA cream (lidocaine 2.5% and prilocaine 2.5%, Astra Zeneca Inc., Sweden) was used to ease venous cannulation. HR, noninvasive arterial blood pressure, blood oxygen saturation (SaO2), electrocardiogram (ECG), and bispectral index (BIS) were monitored. After preoxygenation via face mask, anesthesia was induced with propofol 2–3 mg/kg and inhalation of 6 vol% sevoflurane. After the pupils were fixed, the laryngeal mask airway (LMA) was inserted and the ilioinguinal/iliohypogastric nerve block was performed by ultrasound guidance to relieve postoperative pain. Anesthesia was maintained with 2-3 vol% sevoflurane to maintained BIS from 40 to 60 and retaining spontaneous respiration. After the vital signs were stable, study groups received a rapid bolus injection of different doses of DEX (Jiangsu singch pharmaceutical co., LTD) at a rate of less than 5 s, while patients in the control group received saline in an equal volume. When the surgery was completed, every one of the patients was moved to the post-anesthesia care unit (PACU), and the children naturally regained consciousness.

### Data collection

HR, mean blood pressure (MBP), and SaO_2_ were recorded immediately before the study drug injection (baseline), every minute for 5 min thereafter, and at the time points of the skin cut and hernial sac pull. EA was assessed by the study staff upon arrival in the PACU using the Pediatric Anesthesia Emergence Delirium (PAED) scale (0–20 scale), with a score of > 12 considered to be a diagnosis of EA [12]. Propofol 1 mg/kg was administered via IV for EA if the patient was determined to be pain-free and when the parent or caregiver was unable to comfort the child. Pain was evaluated using the Modified Children’s Hospital of Eastern Ontario Pain Scale (m-CHOPES), and intravenous fentanyl 0.5 μg/kg was administered if the score was≥7. The time of operation and awakening, and any side effects, such as respiratory and cardiovascular depression were recorded. Any supplementary postoperative drugs were also recorded. Finally, patients with an Alderete score ≥ 9 were sent back to the ward.

### Statistical analysis

All of the data are presented as mean ± standard deviation (SD), median (interquartile range), or number (%), as appropriate. The measurement data was analyzed by Shapiro-Wilk. The normal distribution data which satisfied the homogeneity test of variance was tested by ANOVA, if not, by Kruskal-Wallis. The abnormal distribution data was analyzed by Kruskal-Wallis to compare among groups. Pearson’s chi-squared test was used to assess the comparison among groups in count data. The repeated measurement data (MAP and HR) was analyzed by two-way ANOVA, and the comparisons of intra-group and among groups were analyzed by bonferroni. Data were analyzed using SPSS software (SPSS 16.0, SPSS, Inc., Chicago, IL, USA). A *p* value of less than 0.05 was considered to be a significant difference.

## Results

There were 100 pediatric patients who were enrolled and randomly divided into five groups. As seen in Table 1, there were no significant demographic and surgical characteristic differences among the groups.

**Table 1:** Demographic and surgical characteristics of patients

Variable	Group D_1_	Group D_2_	Group D_3_	Group D_4_	Group D_5_
Age, median (IQR), yr	4 (4–5)	5 (3–6)	4 (3.5–5)	4 (3–4.5)	4 (3–5)
Male/Female, No./No.	17/3	17/3	16/4	16/4	16/4
Weight, median (IQR), kg	17.0 (16.0–20.0)	18.3 ± 3.95	17.0 (15.0–17.75)	16.4 ± 3.28	18.5 ± 3.76
Duration of surgery, median (IQR), min	14.4 ± 5.95	11.5 (10.0–14.5)	12.0 (9.5–14.0)	11.0 (9.5–20.5)	11.5 (9.0–13.0)

There were no statistically significant differences (*P* > 0.05) among the groups

The incidence of EA was significantly lower in groups D_4_ and D_5,_ and a trend of fewer was observed in group D_2_ and D_3_ (Table 2).

**Table 2:** Incidence of Emergence Agitation (PAED > 12)

Groups	Number	Incidence (%)	*P* value^*^
Group D_1_	6	30%	
Group D_2_	1	5%	*P* = 0.096
Group D_3_	1	5%	*P* = 0.096
Group D_4_	0	0%	*P* < 0.001
Group D_5_	0	0%	*P* < 0.001

^*^Compared with Group D_1_

There were no significant differences in the basal HR among the five groups. In group D_1_, there were no significant differences among the basal HR and every minute for 5 min thereafter, but the HR at the point of the hernial sac pull was increased significantly compared with the baseline. In group D_2_, the HR was decreased at 1 min compared with the baseline, and then returned to the baseline at 2, 3, 4 and 5 min, and the points of skin cut, but the HR at the point of the hernial sac pull was increased significantly compared with the baseline. In group D_3_, the HR was decreased at 1, 2,3 and 4 min compared with the baseline, and the HR returned to the baseline at 5 min, the points of skin cut and hernial sac pull. In groups D_4_ and D_5_, the HR was decreased at 1, 2, 3, 4 and 5 min compared with the baseline, and the HR returned to the baseline at the time point of the skin cut and hernial sac pull (Table 3).

**Table 3:** Heart Rate Changes

Groups	Baseline	1 min	2 min	3 min	4 min	5 min	Skin cut	Hernial sac pull
Group D_1_ median (IQR)	97.0^△^ (91.0–103.5)	97.5^#△^ (94.0–103.0)	99.0^△^ (94.0–104.5)	99.0^△^ (93.5–106.5)	99.0^△^ (95.0–107.5)	100.5^△^ (94.5–108.5)	104.5^△^ (95.0–111.0)	112.0^# *^ (104.0–118.0)
Group D_2_ median (IQR)	101.5 (86.5–115.0)	91.0^△*^ (81.5–102.5)	95.0^△^(85.0–106.0)	94.5^△^(85.5–107.0)	98.0^△^(85.0–108.5)	97.5^△^(86.5–108.5)	100.5^△^(90.0–112.5)	103.5^# *^ (94.5–130.0)
Group D_3_ median (IQR)	104.5(91.5–109.0)	88.5^*△^ (79.5–97.0)	93.0^*△^ (85.5–99.0)	95.0^*△^ (85.0–101.0)	97.0^*△^ (86.0–103.5)	97.5^△^ (89.0–105.0)	100.0 (95.0–108.5)	104.5 (93.5–112.5)
Group D_4_ mean ± SD	104.0 ± 10.4	89.7 ± 10.2^*△^	93.6 ± 9.4^*△^	96.6± 7.6^*^	97.5 ± 7.5^*^	97.4 ± 7.5^*^	97.1 ± 9.6	102.0 ± 8.7
Group D_5_ median (IQR)	99.0 (93.5–102.0)	84.0^*△^ (79.5–90.5)	88.0^*^ (86.0–95.5)	89.0^*^ (87.5–96.5)	90.0^*^ (86.5–97.0)	89.0^*^ (86.0–96.0)	88.5 (84.0–97.0)	95.5 (89.0–99.5)

^*^Compared with baseline, ^△^compared with the time point of the hernial sac pull, ^#^ Compared with group D_5_, *P* < 0.05

The minimal HR of groups D_3_, D_4_, and D_5_ were decreased significantly compared with groups D_1_. And the duration time of minimal HR was significantly prolonged in group D_5_ compared with group D_1_. There were no significant differences in occurrence time of minimal HR among groups (Table 4).

**Table 4:** Minimal Heart Rate

Groups	Minimal	Duration time(s)	Occurrence time(s)
Group D_1_	92 ± 12	2.0 (1.0–3.5)	30.0 (20.0–42.5)
Group D_2_	88 ± 16	2.0 (2.0–6.0)	42.0 (39.0–49.0)
Group D_3_	83 ± 11^*^	5.0 (3.0–6.5)	38.5 (35.0–43.0)
Group D_4_	84 ± 11^*^	3.0 (2.0–5.0)	41.0 (34.5–50.0)
Group D_5_	81 ± 8^*^	6.0 (2.0–9.0)^*^	41.0 (32.5–49.0)

^*^Compared with group D_1_ respectively, *P* < 0.05

There were no significant differences in the basal MBP among the five groups. In group D_1_, there were no significant differences among the basal MBP and every minute for 5 min thereafter, but the MBP at the point of the hernial sac pull was increased significantly compared with the baseline. In group D_2_, the MBP was decreased at 2, 3, 4, and 5 min compared with the baseline, and the MBP returned to the baseline at the point of skin cut and hernial sac pull. In group D_3_ and D_4_, the MBP was decreased at 3, 4, and 5 min compared with the baseline, and the MBP returned to the baseline at the point of skin cut and hernial sac pull. In group D_5_, the MBP was decreased at 5 min and the points of the skin cut and hernial sac pull compared with the baseline (Table 5).

**Table 5:** MBP Changes [median (IQR)]

Groups	Baseline	1 min	2 min	3 min	4 min	5 min	Skin cut	Hernial sac pull
Group D_1_	58.0^△^ (56.5–62.0)	58.0 (57.0–62.5)	58.0^△^ (56.0–61.5)	58.0^△^ (56.0–61.0)	57.0^△^ (56.0–58.5)	57.5^△^ (55.0–59.5)	61.5 (56.0–64.5)	64.5^*^ (60.0–69.5)
Group D_2_	60.0 (53.5–66.5)	58.5 (51.5–65.0)	57.0^*△^ (49.5–61.0)	58.0^*^ (50.0–60.5)	57.0^*^ (48.0–60.5)	57.0^*△^ (49.5–60.5)	57.0^△^ (52.0–60.0)	62.0 (55.0–70.5)
Group D_3_	58.0 (55.5–60.5)	58.0 (55.5–61.5)	55.5^△^ (52.0–58.0)	53.0^*^ (51.0–56.5)	53.0^*△^ (51.0–55.5)	53.0^*^ (51.0–56.0)	54.5 (51.0–57.5)	58.5 (53.5–61.5)
Group D_4_	59.0 (55.0–65.5)	59.5 (57.5–67.0)	56.5 (53.0–61.0)	55.0^*^ (51.5–60.0)	53.5^*^ (50.0–58.5)	55.0^*△^ (49.5–59.0)	57.5 (50.5–61.0)	59.5 (56.5–64.0)
Group D_5_	57.0 (54.0–63.0)	61.5^△^ (57.5–68.0)	54.5 (51.0–65.0)	54.0 (49.0–60.0)	53.0 (49.5–60.5)	52.5^*^ (50.0–56.5)	53.5^*^ (49.5–59.0)	52.5^*^ (48.5–59.0)

^*^Compared with Baseline, ^△^compared with the time point of hernial sac pull, *P* < 0.05

There were no significant differences in incidencerate of complementary medicine, adverse events, and bradycardia (we defined bradycardia as HR decrease of ≥30% during the 5-min observation period postdexmedetomidine bolus, compared to predexmedetomidine bolus baseline values). There were eight patients who had a 30% decrease from the baseline of HR, and none of the patients required treatment for bradycardia. Four patients needed propofol to treat EA, and no one needed treatment for bradycardia, pain, or other adverse events, including cough, headache, and vomiting (Table 6). All of the patients with spontaneous respiration could maintain SaO_2_ ≥ 98%.There were significant differences in the recovery time between every two groups (*P* < 0.05) (Table 7).

**Table 6:** Comparison of complementary medicine, adverse events, and bradycardia [No. (%)]

Groups	Complementary Medicine	Adverse events	Bradycardia^a^
Group D_1_	3 (15%)	0	0
Group D_2_	0	0	1 (5%)
Group D_3_	1 (5%)	3 (15%)	3 (15%)
Group D_4_	0	1 (5%)	2 (10%)
Group D_5_	0	1 (5%)	2 (10%)

There were no statistically significant differences (*P* > 0.05) among the groups

^a^Bradycardia was defined as HR decrease of ≥30% during the 5-min observation period postdexmedetomidine bolus, compared to predexmedetomidine bolus baseline values

**Table 7:** Recovery time (mean ± SD)

Groups	Recovery time (min)
Group D_1_	21.3 ± 7.1
Group D_2_	30.3 ± 8.6
Group D_3_	40.7 ± 8.2
Group D_4_	49.5 ± 6.4
Group D_5_	60.4 ± 21.1

There were significant differences in the recovery time between every two groups (*P* < 0.05)

## Discussion

Even without a pediatric label, additions to the literature on pediatric applications of DEX have risen in the last few years [4]. DEX has numerous properties that make it beneficial as a sedative and anesthetic; it has been documented to sedate in a manner that is similar to natural sleep, and it is used as an anxiolytic, analgesic, and sympatholytic [1, 2, 13]. In addition, it has an anesthetic-sparing impact with negligible respiratory depression, and it can be used to avert and treat postoperative EA [13]. The hemodynamic reactions of DEX are related to the rate of infusion and dose [5]. Although it is recommended that DEX is infused as a bolus over 10 min, the BP and HR response are still present [2, 14]. As doses of DEX get larger, hypotension and transient hypertension are greater. While there are many clinical encounters in the rapid bolus injection of DEX, there is minimal efficacy data that are accessible [11, 12, 15].

In our study, we found that 0.75 and 1.0 μg/kg DEX rapid infusion could significantly reduce the incidence of EA, while 0.25 and 0.5 μg/kg DEX had the fewer trend. Hauber JA et al. [12] had found that 0.5 μg/kg DEX rapid infusion could reducing the incidence of EA. The difference may because different subjective evaluation of the score of PAED and the smaller sample size. In addition, 0.5, 0.75 and 1.0 μg/kg DEX could reduce the surgical stress of the hernial sac pull. As the dosage increased, the recovery time was significantly prolonged, but there were no significant differences in the incidence of total adverse events. EA is a traumatic experience for children and their parents, and it can result in injury [16]. DEX has been well reported as an effective agent for EA, and the rapid bolus administration of DEX for the treatment of EA is more practical and timely than a 10-min infusion.

DEX could significantly decrease HR and MBP. The minimal HR was significantly lower after the administration of DEX in the dose of 0.5, 0.75 and 1 μg/kg, and the duration time was significantly prolonged after 1.0 μg/kg DEX infusion, but no one needed treatment. In human and animal models, DEX has a biphasic blood pressure impact: a preliminary but transitory elevation in blood pressure accompanied by a long-lasting hypotensive effect that is induced by peripheralα_2_-adrenoreceptor and then central α_2_-adrenoreceptor stimulation [11, 12, 17]. Therefore DEX should be administered as a loading dose during a 10-min period to diminish the dose-dependent, biphasic, hemodynamic reaction. In our study, hypertension was not seen, this could have happened since the rapid infusion of DEX eradicated the time differential between the stimulation of the peripheral and central α_2_-adrenoreceptor.

The general agreement seems to be that DEX is linked to minimal respiratory depression [1, 5, 17, 18]. Our study found that the rapid injection of DEX can cause a decrease in tidal volume, but it does not affect SaO_2_ without special handling. This may because deep sedation and oral/pharyngeal anatomic frequently happened in deep sleep after the administration of DEX.

## Conclusion

In conclusion, rapid infusion of DEX at dose of 0.75, and 1.0 μg/kg DEX could prevent EA. Even though the use of DEX resulted in transient decreases in HR and MBP, the drug is well-tolerated in pediatric patients. Our study provides a new clinical application for the rapid intravenous injection of DEX for the prevention and treatment of EA.

## Abbreviations

BIS: Bispectral index
DEX: Dexmedetomidine
EA: Emergence agitation
ECG: Electrocardiogram
HR: Heart rate
IV: Intravenous injection
LMA: Laryngeal mask airway
MBP: Mean blood pressure
m-CHOPES: Modified Children’s Hospital of Eastern Ontario Pain Scale
PACU: Post-anesthesia care unit
PAED: Pediatric Anesthesia Emergence Delirium
SaO_2_: Blood oxygen saturation
SD: Standard deviation
